# An Analysis of Five Hundred Cases of Skin Diseases

**Published:** 1877-10

**Authors:** W. J. Maynard

**Affiliations:** Chicago


### AN ANALYSIS OF FIVE HUNDRED CASES OF SKIN DISEASES.

By W. J. Maynard, M. D., Chicago.

(Condensed from a Report on Dermatology, to the Chicago Medical Society, May 21st.)

In the past too little attention has been paid by the teachers of medicine to the subject of Dermatology. I was once told, that in treating cutaneous diseases, it was only necessary to remember that, "Skin diseases could be divided into two classes, specific and non-specific. For all non-specific cases give arsenic for a cure, and for all those that have a specific origin, give iodide of potassium." If so concise and brilliant a manner of inviting attention to this large field of practical medicine is generally adopted in the teachings of others, it is not at all strange that this subject is so much neglected, and a large majority of college graduates kept in profound ignorance of a department of medicine, that they, at least, should have a general idea of. This work, however, has been pushed on more vigorously, and thanks to those who have labored the most industriously in this field, we find that in a few years rapid advancement has been made towards greater perfection; and while we find to-day that dermatology is in a very flourishing condition, yet from the increased attention paid to it in every direction, we must expect much more brilliant results in the future, and truths promulgated that will not be merely the results of empirical teaching, but rather those that emanate from thorough investigation and scientific pathological research.

In a report to the State Medical Society of Kentucky, Dr. Yandell makes, as a basis of his report, that chronic skin diseases have their origin in the strumous diathesis, while acute diseases have a malarial origin. He says, also, that struma may give evidence of its active existence at birth, or remain many years latent in the system; bad hygienic surroundings, depraved habits, exposure to changes of temperature, bodily injuries, and acquired diseases, are the most usual excitants of its development into activity. Malaria is a subject on which there is a wide range of opinion, and concerning which but little is definitely known, whether it arises from the emanations of living or decaying plants, or has its origin in microscopic vegetable organisms, or due to certain changes in the constituent elements of the atmosphere, or is chargeable to the excess or deficiency, or change, of some normal constituent of the blood, he cannot discuss, but holds that it is a prolific source of skin disease. Whether or not there is a malarial diathesis, as has been suggested, there is such a thing as latent malaria, and this, like struma, can be germinated, incubated, ignited or crystallized by changes of temperature, dentition, physical injuries, improper, excessive, or insufficient food.

To these, also, we would add the syphilitic diathesis as a very active agent in the production of these lesions; and while we cannot go as far as he and assert that skin diseases have their direct origin in certain diathesis, yet we would rather regard them as part or modifying causes, and as long as they exist have a tendency to perpetuate or intensify the existing disease.

The more experience we have in the treatment of these maladies, the more we are convinced that such conditions do lie in the back-ground, particularly in young life, and without the aid of remedies that are directed to these peculiar conditions, we find that chronic skin-diseases are exceedingly obstinate, if not incurable. Thus, in the treatment of cutaneous disorders, must we be very thorough in searching for the cause of disease, and although we see many that have simply a local origin, a large majority that come under our observation have their origin from within the body, and while it is very essential that we should direct our attention closely to the local manifestations, yet for the permanent cure of the disease, we must remove that cause, whether this be due to some organic disease of internal organs, to mal-nutrition, to blood alterations, from bad assimilations, or due to degenerative tissue changes. Thus Piffard, in speaking of the rheumides (in this class he puts eczema, psoriasis, pityriasis, etc.) says, "it may be formally stated that the affections pertaining to this diathesis are all probably due to an accumulation in the blood of an excess of certain excrementitious substances, and presumably those that are efficient in the causation of gout and rheumatism; the blood is subalkaline, that is, deficient in alkalies. The accumulation in the blood of these excreta is due to either deficient kidney action, or these substances are produced in excess. This excess is due either to over-supply of albuminoid food, the surplus not being thoroughly oxidized, or there is a failure on the part of the oxidizing processes to fully complete the changes. Murchison says, "there are strong reasons to believe that the liver is the organ more particularly at fault in this connection. The principal indications then to be filled are, to depurate the blood by calling into action the kidneys, bowels and skin, and promoting oxidation by taking away excessive amount of meat, if this condition is due to incomplete oxidation of an excessive amount of albuminoid ingesta. Increase oxidation also by preparations of iron, chlorate of potash, etc., stir up the liver, encourage out-door exercise, good ventilation, bathing, and diet."

These conclusions, although of the utmost importance in treatment, cannot now be further spoken of, for the reason that the main object that I have in view in presenting this article is to give, in as brief a manner as possible, an analysis of 500 cases of skin diseases that I have met with during the last two years (the exact number is 547), and wish to invite attention to some practical methods of treatment that experience has shown to be of value in these disorders. It is simply impossible in dispensary practice to carry out a thorough line of treatment, on account of the ignorance and carelessness of the patients and their manner of living, and then, too, many not being able to leave their daily employment, are irregular in their attendance, and negligent in obeying instructions, and by these means prolong a disease that might have been of short duration. However, in spite of all this, I have seen the most gratifying results in treatment, and in the management of these cases I claim nothing that is particularly original, but, rather, have followed the teachings of the more modern school of dermatology. This analysis shows that eczema stands first in point of frequency, occurring 158 times in the 547 cases. By comparison, I find that this ratio is about the same as that given by other observers. In the management of these cases of eczema I always first look for a cause, and in most instances find that debility, over-work, stomach derangements, and local irritation lie at the root of the trouble, and endeavor as soon as possible to do away with these conditions. Then, in some we find we have to do with gout, rheumatism, and deficient kidney action, and in young life we have hereditary and diathetic influences. Thus, the causes being multiple, it follows that each case must have its own peculiar treatment, while the local treatment must vary according to the particular stages of the disease. Thus, in the more acute stages, where the exudation is great, I rely upon the absorbent powders, as oxide of zinc and starch, equal parts, to which a little camphor may be added; another has been used with benefit, as liquor plumbi subacetatis ʒij, tinct. opii ʒiv, to twelve or sixteen ounces of water. In the subacute stage, where the exudation has in a great measure ceased, and where there may be slight scaliness, I prefer the ointments, as the benzoated oxide of zinc, or what answers better in my hands, a mild mercurial, as the ammonio-chloride five to ten grains to the ounce. Where there is no discharge, but dry patches with more or less infiltration, I rely upon tarry preparations, as oil of cade ʒij to the ounce, or the strong alkalies, as green soap, either alone or mixed with alcohol, or what can be used in its place with good results caustic potash two grains to ℥ss to an oz. f. In private practice I rarely use lard as an excipient, for the reason that it soon becomes rancid and does harm. In its place I usually prescribe cosmoline, vaseline, or rose ointment. In eczema of the head in children, I always direct that the thick crusts be at first removed by a poultice, and then do not allow them to use it again, or wash the head, but prevent the re-accumulation of scales by soaking the head in cod liver oil, afterwards using a mercurial or tarry ointment according to the conditions present, the head at the same time covered with an oil-silk cap. Cod liver oil also is freely given internally, either with or without the syrup of the iodide of iron.

The next in order of frequency come the syphilodermata, ninety-four in number. In these cases all primary and secondary forms of the disease were treated with preparations of mercury, the most effective of which was the bichloride. In all later forms of the secondary stages, the best results were obtained from the so-called mixed treatment, that is a combination of mercury with iodide of potassium.

One of the most obstinate and unsatisfactory diseases that we we are called upon to treat is acne. Thirty-one cases are recorded. Although this is generally regarded as a disorder of adolescence, yet I have seen it frequently in those who were beyond this period of life, and think that in a majority of my cases there was associated more or less stomach derangements, and constipation, and uterine troubles. These causes were removed as far as possible, and at the same time the patients were directed to bathe the face night and morning in hot water, and apply lotions to the parts. If I have to deal with the tuberculous variety, I generally use sulphur in some form, as the iodide of sulphur ointment, or, what is better still, equal parts of sulphur, alcohol, glycerine, and water. I have used in some cases, with much benefit, a lotion of borax, bichloride of mercury, glycerine, and water. Where these failed to do good, good results have been obtained from a lotion of sulphate of potash, sulph. zinc, glycerine, and water. In acne rosacea caustics are necessary. Neumann extols one part of carbolic acid to three or four of alcohol.

In this list are found thirty-seven cases of psoriasis. Great care is sometimes necessary to distinguish the non-syphilitic from those of a syphilitic origin in this class, for the reason that the usual diagnostic marks and a syphilitic history are sometimes absent. In acute cases of psoriasis I am in the habit of giving alkaline diuretics freely. A large majority of these cases were chronic, and were treated with carbolic acid internally in four drop doses gradually increased. Some of these patients made better progress with a combination of carbolic acid and arsenic. Locally, the parts were rubbed with mercurial, tarry, or alkaline ointments, according to the case. I have a patient under my care at the present time who has had the disease for twenty-four years, and for the first time is improving rapidly under a treatment of carbolic acid and Fowler's solution of arsenic, with frictions of green soap and alkaline baths. In a recent number of the London Lancet, Dr. Cuttle recommends very highly in psoriasis, a solution of India rubber which he paints over the affected patches, after having removed the scales, renewing the operation from time to time in order to maintain an impervious coating. He cites fifty cases treated in this manner, and with great success. This is really a modification of the plan adopted and extolled by Hebra on the continent, and McCall Anderson, of Glasgow, and I mention it in this connection, for it is really an improvement, inasmuch as it is much less expensive, and fills the same indications.

There were fifty-one cases due to the animal parasites; thirty-nine of these were from pediculi, and are called phthiriasis.

It is not at all necessary, in a disease of this kind, to search the clothes for the offending parasite, as the hæmorrhagic mark that it leaves upon the skin is quite characteristic, and when discovered is absolutely diagnostic. All that is necessary in treatment is to thoroughly bake the clothes and apply ammonio-chloride of mercury ointment to the skin. Twelve of these were due to the acarus scabiei, and were all treated locally with sulphur ointment, or what I found a more speedy method of cure was an ointment of sulphur ʒss, grs. v. of the white precipitate with five to six drops of carbolic acid to the oz.

Twenty-five cases of urticaria recorded were mostly chronic, and were treated locally by alkaline and sedative lotions, and by removing the cause wherever it could possibly be found. There were found twenty-three diseases due to the vegetable parasites, comprising nearly all of the different varieties, of which there were ten cases of tinea circinata, three of tinea tonsurans, six of tinea versicolor, four of tinea sycosis. Recognizing the fact that the disease depended chiefly upon local irritation, the treatment in these cases was entirely local, and consisted either in white or red precipitate ointments, lotions of the bichloride of mercury, or a strong solution of borax. A great deal has been said lately in the journals of the influence of goa powder over these diseases, and as a parasiticide is said to be more certain in its action than anything yet known. This goa powder is a vegetable substance, and is the product of some unknown plant. It comes from Brazil, where it is known as araroba. Its chief active properties are supposed to be due to chrysophanic acid.

There were twenty-two cases of herpes. Among this number are found all the different varieties, with the exception of herpes iris. which I never have seen. Among my eighteen cases of erythema were found all the different varieties of this disease, and in nearly all there was more or less disturbance of the digestive functions. One case of erythema nodosum was easily cured by quinine, where other remedies had failed to do good.

There were seventeen cases of impetigo, six of which were of the so-called impetigo contagiosa. In these diseases tonics and cod-liver oil are always indicated, with removal of the scabs, and topical application of glycero-tannin, or a mild mercurial ointment. Eighteen cases of pruritus are recorded in different parts of the body, and depending upon different causes, which in most cases must be removed before the trouble will cease.

There are recorded in this list three cases of purpura; very little is known as yet in regard to the true pathological condition present in this disease, and consequently the treatment has generally been unsatisfactory. My first case was treated by astringents, as almost universally recommended by the books, as solutio ferri persulph., turpentine, etc., but without the least success. My failure in this case induced me, in the next two, to resort to ergot, and obtained the very best results, both cases that had been existing for some time, yielded very easily to its influence. I used the fl. ext. in teaspoonful doses three times a day. The decided power it has over the involuntary muscular fibres, and the manner in which it arrests hæmorrhage in other affections will account for its speedy action in these cutaneous hæmorrhages.

Eight cases of lichen are recorded, of which six were of the simple variety, two of lichen pilaris, and one answered well to that form of disease the lichen ruber of Hebra. They were all treated by sedative lotions and tonics.

Thus briefly have we selected a few of the more prevalent forms of the recorded five hundred cases, and have given a general idea of their management. The remainder of the list comprises three forms of skin diseases that we occassionally meet with, and as their history may not be particularly intresting to all, I will not allude to them further than to mention that there are eight cases of lupus recorded, five cases of pityriasis, and three of ecthyma. Seborrhoea occurred four times, and erysipelas three; four cases of eczema marginatum. There are two cases of ichthyosis and two of chloasma. One case of that rare form of disease prurigo; only one of pemphigus, and one of hydroa. Alopecia areata was seen only once, and likewise keloid, xerodema, and elephantiasis arabum.

